# A critical review of scoring options for clinical measurement tools

**DOI:** 10.1186/s13104-015-1561-6

**Published:** 2015-10-28

**Authors:** Maria Laura Avila, Jennifer Stinson, Alex Kiss, Leonardo R. Brandão, Elizabeth Uleryk, Brian M. Feldman

**Affiliations:** Department of Pediatrics, The Hospital for Sick Children, University of Toronto, Toronto, Ontario Canada; Lawrence S. Bloomberg Faculty of Nursing, University of Toronto, Toronto, Ontario Canada; Child Health Evaluative Sciences, The Hospital for Sick Children, University of Toronto, Toronto, Ontario Canada; Department of Research Design and Biostatistics, Institute for Clinical Evaluative Sciences, Sunnybrook Health Sciences Centre, Toronto, Ontario Canada

**Keywords:** Formative, Reflective, Score, Scoring, Measurement

## Abstract

**Background:**

The aim of this paper is twofold: (1) to describe the fundamental differences between formative and reflective measurement models, and (2) to review the options proposed in the literature to obtain overall instrument summary scores, with a particular focus on formative models.

**Methods:**

An extensive literature search was conducted using the following databases: MEDLINE, EMBASE, PsycINFO, CINAHL and ABI/INFORM, using “formative” and “reflective” as text words; relevant articles’ reference lists were hand searched.

**Results:**

Reflective models are most frequently scored by means of simple summation, which is consistent with the theory underlying these models. However, our review suggests that formative models might be better summarized using weighted combinations of indicators, since each indicator captures unique features of the underlying construct. For this purpose, indicator weights have been obtained using choice-based, statistical, researcher-based, and combined approaches.

**Conclusion:**

Whereas simple summation is a theoretically justified scoring system for reflective measurement models, formative measures likely benefit from the use of weighted scores that preserve the contribution of each of the aspects of the construct.

**Electronic supplementary material:**

The online version of this article (doi:10.1186/s13104-015-1561-6) contains supplementary material, which is available to authorized users.

## Background

From a holistic perspective [1], measurement has been described as an empirical process of “assigning numbers to objects or events according to a rule” [2] as well as an intellectual activity of “giving meaning to the theoretical variables”.

A measurement model describes the relationship between a *construct* and its *indicators* [3]. A construct can be defined as an abstract phenomenon of interest, and indicators as the observable elements used to assess this construct [3, 4]. For example, *melancholia* is a construct, and “depressed mood”, “tiredness”, and “sleep disturbance” are some of the indicators used to assess melancholia [5].

*Psychometrics,* or the study of the theories and techniques concerned with the measurement of mental manifestations and phenomena [5, 6], has influenced the design of the measurement tools used in social and health sciences for more than a century [7]. However, it has been stated that “the foundations of psychometric theory are full of theoretical tensions and fissures that mostly go unnoticed in the daily activity of test construction and use” [8].

One of these fissures, which has received increasing attention for the past three decades, is the meaning of indicators in a measurement model.

In general, instruments developed under psychometric theory (typically for the measurement of mental characteristics [9] ) aim to capture the entirety of an underlying construct [10, 11], for example *melancholia*. A battery of homogeneous and positively intercorrelated indicators are thus selected because they all reflect the construct being measured [12]—for example, “tiredness” and “depressed mood” may be items of a melancholia scale. As defined by Fayers, homogeneity refers to the fact that the indicators are expected to equally tap into the same construct, [12, 13]. However, the assumption that the indicators used in a measurement tool are homogeneous and positively intercorrelated does not hold true in some cases [14]. For example, the construct *life stress* can be measured by indicators such as “job loss”, “divorce”, and “death in the family” [7]. In contrast to the indicators used to assess melancholia, each of these indicators can be seen as a more distinct and unique aspect of the construct.

It was in the social sciences that indicators that were not necessarily homogeneous and positively intercorrelated were first formally used in their measurement tools (in view of the specific characteristics and different nature of the constructs studied in this field). These indicators were termed “cause/causal” indicators, as opposed to “effect” indicators, which prevail in the psychometric tradition [15, 16]. Indeed, in the 1960s, Curtis et al. noted that the traditional psychometric approach was not fully appropriate to measure aspects of research in sociology—in which there were valid but unrelated or even inversely correlated indicators of the same construct [16]. The differences between the types of indicators were further explored in the field of sociology by Hubert Blalock Jr., who was the first to describe the distinction between cause (formative) and effect (reflective) indicators [15, 17]. Similarly, in the field of marketing, cause/causal and effect indicators [4] were adopted and referred to as “formative” and “reflective”, respectively. More recently, the terminology of formative and reflective indicators was introduced into the health sciences in the 2000s by the work of Fayers and Hand [12] for the measurement of Quality of Life (QoL).

Whereas reflective models represent the classical concept of measurement used in psychometrics [18, 19], formative measurement models were proposed as an alternative to measure constructs for which the application of a traditional reflective measurement approach would have violated its theoretical foundation. Formative models apply to constructs that are represented by different facets (domains or dimensions) [11], so that constructs in formative models are not unidimensional, but rather result from the combination of heterogeneous indicators [7, 20].

Understanding the difference between reflective and formative measurement models is highly relevant during the development of a measurement tool. The choice of the scoring method is an important step in the development of an instrument and should be consistent with the choice of a measurement model. The scores of a tool are in fact an essential component of the validity of the instrument. Messick defined validity as a property not of the test, but of the meaning, interpretation, and implications of the test scores [21]. Therefore, decisions regarding the choice of a scoring system are deeply attached to the nature of the construct, and have implications for the validity of any instrument. Researchers developing a measurement tool should be aware of the different perspectives regarding measurement models and their impact on scoring systems, in order to decide which approach better corresponds to his or her objective.

The objective of this paper is to offer a brief summary of the fundaments of formative and reflective measurement models, and to review the different approaches used to obtain summary scores that have been proposed in the literature.

This review is particularly intended for the clinical researcher and practitioner since it focuses on the less traditional formative models, which may be of more value in the clinical setting.

## Methods

An extensive literature search was conducted with the assistance of an experienced research librarian to identify technical papers or manuscripts that have described and/or discussed the issue of formative and reflective models. The search strategies and terms are shown in Additional file 1.

The searches were run using (1) the OvidSP search platform using the following databases: MEDLINE, EMBASE, and PsycINFO; (2) the EBSCOHost search platform using the following database: CINAHL and (3) the ProQuest search platform using the following database: ABI/INFORM to include articles indexed as of February 25, 2013. The references of identified articles were screened for additional studies.

All articles discussing conceptual issues related to scoring methods in formative and reflective models were included in this narrative review in order to address the second objective.

It is worth noting that although the literature was searched in a systematic manner and all the papers matching the inclusion criteria were retrieved, the theoretical and abstract nature of the subject of the present study did not allow following some of the usual steps involved in a systematic review. For example, the PRISMA checklist and tools for assessing risk of bias were developed to assess health-related interventions or outcomes, and cannot be used in the setting of our study. For this reason, the term “systematic” was avoided when describing the methodology followed herein.

Ethics approval was not required for this study. All the data collected are presented in the manuscript.

## Results

### Part I: Theoretical foundations (fundaments) of reflective and formative measurement models

The distinction between formative and reflective models is not only of theoretical nature; it has implications in the design and validation of measurement instruments [22].

The *reflective measurement model* stems from classical test theory (CTT), and is the basis for factor analysis [23]. According to CTT, the observed score (O), or test score obtained from a measurement instrument, comprises two parts: the true underlying score (T), which represents the hypothetical unobservable value that a subject has for a construct, and random error (E), which is the part of the observed score that can be attributed to measurement error [24]:

1 $${\text{O}} = {\text{T}} + {\text{E}}$$

Consistent with CTT, the observable indicators y_i_ in reflective models are considered to be a manifestation of a hypothetical construct (or latent variable) η.

2 $${\text{y}}_{\text{i}} = \uplambda_{\text{i}} \upeta + \upvarepsilon_{\text{i}}$$

where λ represents a coefficient capturing the effect of the construct η on an indicator y_i_, and ε_i_ represents the measurement error for y_i_ [3]. Thus, according to this regression model, the observable indicator y_i_ is a function of the latent variable η and of measurement error ε_i_. Variation in the scores of the indicators is assumed to be a function of a true score plus measurement error at the indicator level [25, 26] (Eqs. 1, 2).

**Fig. 1:**
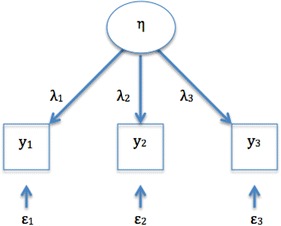
Direction of causality in reflective models. η, represent the construct; y_i_, the observable indicators; λ, the coefficients linking the construct η to the indicators y_i_; ε, the error term associated with y_i_
- *Characteristics of indicators and indicator intercorrelation* Because the underlying latent variable or construct influences the indicators, the indicators are intercorrelated. Thus, covariance among indicators reflects variation in the latent variable. Moreover, it is expected that all the indicators will have a high positive correlation and high internal consistency. Therefore, indicators can be interchanged, and elimination of an indicator from the measurement model should not change the meaning of the construct [20, 27–31].
- *Measurement error* Reflective models include an error term that, as shown in Eq. (2), is associated to each indicator. Edwards defines this term as “uniqueness” of the indicator, which combines measurement error and indicator specificity [11].
- *Indicator relationship with construct antecedents and consequences* The meaning of a construct depends not only on its relationship with its indicators, but also on its relationship with other constructs to which it is connected through a complex network of interlocking laws, known as a nomological network [1]. These laws can link constructs to other constructs (e.g., the construct of self-esteem to the construct of emotional stability), constructs to observed measurement (the construct of self-esteem to the measurement of positive attitude towards self), or observed measurement to observed measurement (the measurement of positive attitude towards self to the measurement of being satisfied with self) [32]. The nomological network helps define a theory, where the meaning of a construct is dependent on its antecedents, or causes, and on its consequences, implications or results. Because the indicators of a reflective model are assumed to be interchangeable, the theoretical implication is that they have a similar relationship with the antecedents and consequences [20].

Coltman et al. distinguish reflective and formative models based on theoretical and empirical features [27]. Following are the characteristics of reflective models:

- *Nature of the construct* The underlying latent construct is thought to exist separately from its measures [11]. This concept is akin to “philosophical realism”, and it will be further examined in the discussion section.
- *Direction of causality* The direction of causality flows from the construct to the indicators (Fig. 1). A critical aspect of these models is that an *underlying construct influences its indicators* [28], and changes in the underlying construct are *reflected* by simultaneous changes in all the indicators.

*Formative models* abandon the idea of a single latent variable causing all the indicators, assuming, essentially, the opposite—that in certain cases the indicators jointly determine the meaning of the construct. Therefore, this model has *indicators x causing the underlying construct η* [7, 33]:

3 $$\upeta = \upgamma_{ 1} {\text{x}}_{ 1} + \upgamma_{ 2} {\text{x}}_{ 2} + \cdots + \upgamma_{\text{i}} {\text{x}}_{\text{i}} + \upzeta$$

where γ represents the effect of the indicator x_i_ on the underlying construct η. ζ is a disturbance term that represents all the remaining causes of the construct that are not explained by the indicators [3]. As opposed to Eq. (2), the construct η is the dependent variable, which is explained by its indicators x_i_.

**Fig. 2:**
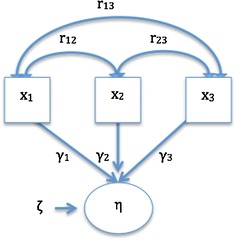
Direction of causality in formative models. η, represent the construct; y_i_, the observable indicators; ϒ_i_, the coefficients indicating the contribution of x_i_ to the construct η; ζ, the disturbance term; r, the correlations between x_i_
- *Characteristics of indicators and indicator intercorrelation* It is a change in the indicators that determines a change in the value of the underlying construct [20]. However, a change in *one* indicator is not necessarily accompanied by a change in *all* indicators. A typical example of this model is socio-economic status (SES) [34], which can be defined as a combination of occupation, education, residence, and income: If one of the indicators changes, SES changes, but if SES changes, not all indicators will necessarily change.
  There are no specific expectations about the correlations between/among formative indicators: they may display positive, negative, or zero correlation. Positive correlations may exist only because the indicators are capturing the same concept. Determination of internal consistency is therefore not appropriate, and indicators are not interchangeable as each captures a specific aspect of the construct. Therefore, elimination of one indicator carries the risk of changing or affecting the meaning of the construct [3, 14, 20, 27, 30].
- *Measurement error* Formative models do not incorporate measurement error, but they specify a disturbance term at the construct level which, as noted above, represents all the aspects or determinants of the construct that have not been specified [35].
- *Indicator relationship with construct antecedents and consequences* Because of their potential heterogeneity or diversity, indicators of formative models do not necessarily have the same relationship with construct antecedents and consequences [27]. Each indicator of formative models conveys unique and distinct information. Importantly, this difference between measurement models as regards the relations of the construct with antecedents and consequences should affect the approach to obtain the overall summary of an instrument, as will be discussed in the next section.

Based on the criteria delineated by Cotlman et al. [27], the characteristics of formative models are as follows:

- *Nature of the construct* The construct being measured is defined (*formed*) according to the indicators the researchers select to measure it.
- *Direction of causality* The relationship flows from the indicators to the construct, as shown in Fig. 2.

### Part II: Scoring methods in reflective and formative models

Results of the search: 1104 citations were retrieved (Additional file 1). References were saved in an EndNote X6 library, which was used to identify 357 duplicates. The remaining 747 unique references were reviewed against the inclusion criteria; 136 were retrieved in full for assessment. Finally, 23 unique references offered methodological perspectives on the approach to obtain summary scores in formative and reflective models, and constitute the core of this review (Additional file 2).

Synthesis of results: In reflective models, the underlying construct determines the score of each indicator [36], whereas in formative models, the indicators are the determinants of the underlying construct. This difference in the relationship between indicators and construct influences the methods used to obtain an overall score, and applies to instruments that consist of more than one indicator (i.e., multi-indicator or multi-item instruments) [37]. As most available scaling guidelines and textbooks refer to the development of reflective models, we will pay special attention to the methods pertaining to formative models. The scoring concepts that apply to reflective models are explained briefly to better understand the theory behind score generation in formative models.

#### Reflective models

According to measurement theory, in reflective measurement models the underlying construct contributes to each indicator, and each indicator is an estimate of the construct. As such, reflective models are most frequently scored by means of simple summation [12, 14, 37]. Summation is one of the most commonly used techniques in social sciences, and its invention is attributed to Rensis Likert [24]. The theoretical foundation for summation comes from CTT. As can be seen in Eq. (1), the observed score in CTT is considered to be a function of the true score plus random error, which has a normal distribution with a mean of 0. Hence, with the summation of several indicators, error will tend to average to 0 [4, 24]. Thus, summation of the reflective indicators is considered a sensible method of estimation [22]. In this process, individual indicators are given a score, and the scores are then added up.

Scores of instruments with multiple subscales that use different metrics in each one of the subscales can be transformed (standardized). Hence, standardized subscales and subscales that have the same metrics can also be added up, which implies equal contribution (or weighting) of each subscale.

Indicator weighting is employed to gauge the contribution of each of the indicators of an instrument to the overall score. In order to implement weights, indicator scores are multiplied by a factor and then added up; factors can be either chosen by the researcher (“theoretical” or “judgment derived weights”) or obtained from the beta coefficients in a regression analysis, or from factor loadings in factor analysis (“empirical weights”) [14, 37, 38]. Despite its logical appeal, the use of weights in reflective models has been reported to have little impact on results [12, 14, 22]. This holds true particularly for scales with highly intercorrelated and/or a large number of indicators [14, 37]. The low impact of weighting is not unexpected since, according the underlying theory, indicators should be highly intercorrelated and interchangeably important [38].

Instruments developed using structural equation modeling (SEM) techniques [39], and even those based on modern psychometric methods such as item-response theory (IRT), also use aggregate sum scores. Even though IRT models allow more complex scoring approaches, it remains unclear whether these approaches yield superior results, and summation remains a simple viable method [12]. It is important to note that what CTT and the more modern psychometric methods, including IRT, have in common is that their analyses nearly always assume the use of reflective indicators [17].

Summation is straightforward in scales based on reflective models that capture a unidimensional construct. In these cases, all the items in the scale relate to a single construct and a variation of the global scale score is easily understood to reflect a variation in the underlying construct. Some researchers also advocate for the use of global summed scores in complex multidimensional instruments composed of multiple subscales, particularly when the subscales are highly intercorrelated or when there are concerns about the performance or reliability of a subscale. In such cases, researchers may prefer reporting a total score, since it is based on more indicators [40, 41]. In the context of reflective models, multidimensional instruments are instruments that measure “higher-level” constructs using reflective indicators at all levels. The concepts pertaining to construct structure (i.e., first or second order constructs) [42] are not addressed here, as they are beyond the scope of this work.

Some experts consider that multidimensionality does not necessarily justify the scoring and reporting of subscales, because subscales may not always provide accurate, unique, and reliable information about the corresponding subdimension [40]. In contrast, other experts highlight the interpretational ambiguities that summed scores can create [43] and, therefore, the issues regarding scoring in formative models discussed below may also apply to the scoring of multidimensional scales composed of reflective indicators.

#### Formative models

There is no consensus about the approach to summarize formative instruments. Some researchers consider that formative indicators can be dealt with using simple summation to obtain an overall rating [23, 27, 34, 44]; adding up each indicator in an overall score (simple summation) or obtaining an average score dividing the total score by the number of indicators has been proposed in order to facilitate the use of these instruments in applied research [45].

However, a major concern is that whereas aggregation of indicators achieves the objective of model parsimony, the distinct and unique information each indicator provides can be lost [27]. It is the opinion of some experts that when formative indicators are involved, neither simple summation nor weighted sums are easy to justify, because each indicator refers to a different aspect of the construct [12], and some indicators may be more important than others [37].

In addition to the loss of information, the use of average scores can potentially result in a cancellation effect. Cancellation occurs when there is a high score in one indicator and low scores in the remaining indicators, leading to a lower overall score [12, 46] and obscuring the contribution of indicators that may be of particular relevance. Summation lumps together respondents that have the same overall score, independently of their pattern of indicators [47]. This issue should be considered if discriminating subgroups of patients or respondents is relevant to the objective of the measurement instrument [47].

Howell et al. [23] have further elaborated on the issue of loss of information when adding up formative uncorrelated indicators. The researchers explained that the number of possible combinations of the scores of every indicator in an index (e.g., 5^3^ = 125 in the case of an index consisting of three indicators, each one measured using a 5-point ordinal scoring system) means loss of information, as there are fewer possible overall results when the individual indicator scores are summed (15 in this case). Moreover, each of the possible 125 combinations may be unique, yet this uniqueness is lost by only considering 15 possible values. When the indicators of a model are highly correlated, the number of observed configurations will be substantially smaller because most configurations will be rather homogenous. This is not necessarily the case for formative indicators, and more possible configurations can therefore be expected [23].

Simple summation implies equal weighting. Indices that contain relatively more indicators for one particular aspect of the construct in a formative measure are implicitly weighting that aspect differently [46]. The weights of formative indicators convey information about their relative contribution to the construct [48].

Following, are different weighting techniques reported in the literature in the context of formative measures:

1. *Choice*-*based approach* It has been suggested in the literature that preferences derived from individuals or groups may be particularly important for weighting combinations in formative models [22]. Preference-based methods such as utility analysis and discrete choice experiments, and the Schedule for the evaluation of individual QoL have been reported as weighting techniques for formative models.
  Preference-based methods are based on the judgment of the value that is placed on a particular outcome (e.g., a particular pattern of indicator responses).
  The terms *preference*, *values*, and *utility* are linked to these methods, and though sometimes used interchangeably, according to some, they represent different concepts [49]. “*Preference”* is a more general term that describes the “desirability of a set of outcomes” [50]. According to Drummond et al., “*Values”* refers to the preferences elicited under conditions of certainty and are evaluated with methods such as rating scales (RS) and time-trade-off (TTO). “*Utility”* refers to the preferences elicited under conditions of uncertainty and is measured using methods like standard gamble (SG) [49, 50].
  The three methods, RS, TTO, SG are the most commonly used methods to measure preferences. The basic form of RS uses simple scales asking respondents to rate a given health condition (e.g., from 0 to 10). SG and TTO involve choice, exploring the willingness of an individual to take a risk in order to gain a benefit [51]. The SG technique requires the individual to hypothetically choose between a certainty (e.g., continuing life in the current health state) and a gamble (which has a probability of resulting in perfect health or death). As for TTO, the aim of the choice task is to elicit the amount of time a participant is willing to sacrifice in order to avoid a worse condition (e.g., a worse health state). A number of authors have addressed these techniques in detail [52–54].
  There is a long-standing debate on which method should be used, in view of theoretical concerns regarding the inconsistency of results and the difficulty of some of the tasks. These considerations highlight the complexity of the human judgment process [51, 53]. Furthermore, it is not yet clear whose preferences should be elicited (e.g., for health scales, whether it should be patients/actual users or the general population) [53, 54].
  Regardless of the method the researcher uses to elicit preferences, choice-based techniques are considered to be particularly important for obtaining weights in formative models [22]. According to the results of the present review, two techniques have been used in the context of formative models:
  - Utility analysis has mainly been used in QoL assessment as an alternative to the psychometric approach. According to Lenert et al., utility in this context reflects the willingness of an individual to take risks in order to gain a benefit, and is used as a numeric measure to address significance in a systematic manner, using the judgment of an individual [51]. Multi-attribute utility theory, which has been used in formative models [55], is an extension of the traditional utility theory, and allows quantifying the utility derived from each attribute and combining utilities in a summary measure [56]. For example, this approach was applied to the Health Utilities Index Mark 2 [55].
  - Discrete choice experiment is a preference-based method that derives from behavioral theory, and has been applied in the context of QoL [57]. The premise is that a construct can be described by its attributes (i.e., relevant factors that affect the decisions of an individual [58] ), and the value assigned by individuals to those attributes can be used to elicit the value of the construct [57, 59]. This method can be used to estimate the relative importance or the weights of attributes by using a judgmental task based on paired comparisons [57]. Respondents are requested to choose between paired hypothetical scenarios that compare, for example, attributes related to cancer treatment (e.g., *improvement in survival* and *urinary function*). Each paired comparison combines different levels of the investigated attributes (e.g., improvement in survival *4*, *8*, or *12* *years* and urinary function *unimpaired, somewhat,* or *severely impaired*). Choices are then analyzed using regression methods [59].
  - Schedule for the evaluation of individual Quality of Life (SEIQoL): This method is a quantitative technique that has been used to elicit preferences in health care [54]. It stems from the idea that people define and evaluate the aspects of their lives in different ways, and therefore, they estimate the relative importance of each aspect differently. In short, SEIQoL consists of having respondents nominate the five areas of life that they consider most important, and rate their satisfaction/functioning in each of these areas. Finally, the relative importance, or weight, of each area is determined using the SEIQoL-direct weighting technique – respondents fill in a pie chart in which the weight of each aspect is equivalent to the proportion of each sector of the pie; weights are read on the chart circumference [57, 60].
2. *Statistical approach* Structural Equation Modeling refers to an expanding family of statistical methods that provide a quantitative test for a theoretical model specified by the researcher. It depicts how a set of indicators relate to a construct and how constructs relate to each other using information about their variances or covariances [61].
  The hypothetical relationships that the researcher conceptualizes when specifying a model can be expressed as parameters. (To estimate these parameters, a basic principle states that the number of unknown parameters cannot be larger than the number of pieces of information provided by the variance–covariance matrix. This concept is known as model identification). The problem is that the basic formative model per se is not identified. To achieve identification, it has been suggested that at least two reflective indicators must be added as consequences of the formative construct [42, 62, 63]. When two reflective indicators are added directly to the construct, a multiple indicator multiple cause (MIMIC) model is obtained [11]. Thus, MIMIC models are special cases of SEM proposed to operationalize formative indicators that classically involve reflective indicators *x*_*i*_, directly or indirectly caused by the underlying construct *n* (Fig. 3), as well as formative indicators *y*_*i*_. For example, the formative indicators *task performance, job dedication, and interpersonal facilitation* can be considered different facets of the construct *job performance*, whereas reflective indicators may include indicators such as “overall, this employee performs the job well” or “this employee fulfills job requirements” [11, 42].

**Fig. 3:**
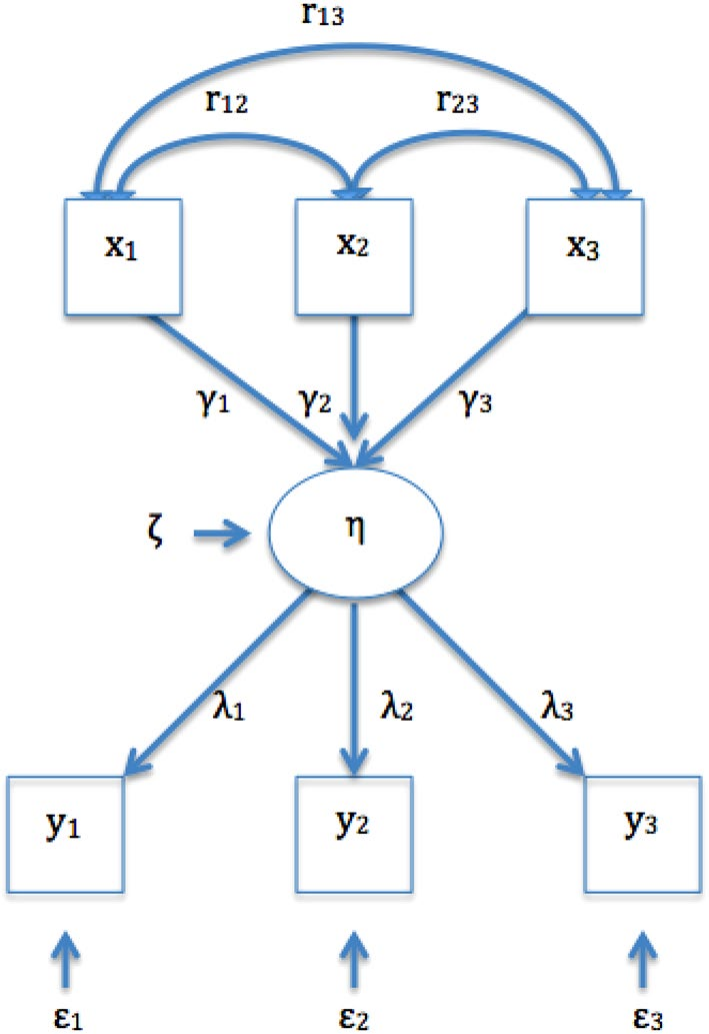
Multiple indicators and multiple causes (MIMIC) models. η, represent the construct; y_i_, the observable reflective indicators; λ, the coefficients linking the construct η to the reflective indicators y_i_; ε, the error term for y_i_; x_i_, the observable formative indicators; ϒ_i_, the coefficients indicating the contribution of x_i_ to the construct η; ζ, the disturbance term; r, the correlations between x_i_
  In MIMIC models, the construct is summarized as the sum of the regression coefficients or betas of its formative indicators (i.e., weighted sum) [12].
  However, the adequacy of adding reflective measures to a model in order to achieve identification, independently of the conceptual relevance and impact of these measures in the construct, has been subject of high controversy for the past years. The central problem is that the meaning of the construct in MIMIC models is now a function of both x_i_ and y_i_. According to Bagozzi, the construct functions figuratively, linking the information contained in x_i_ to that contained in y_i_. This makes the model valuable for the prediction of y_i_ by x_i_, but hinders the possibility of interpreting the construct in a meaningful way [64]. Moreover, the choice of reflective indicators x_i_ can have a profound effect on the construct, because choosing a different set of reflective indicators can substantially alter the empirical meaning of the construct. This issue could create further problems in construct interpretation (i.e., interpretational confounding) [11, 23, 65], which in turn affects the comparability of measurements between/among studies (i.e., generalizability) [65]. All these issues have led experts to challenge the suitability of current approaches to deal with formative models in the context of SEM [11, 66], and to propose alternative models to solve these issues [11, 67]. Hence, MIMIC models should be used with caution in the estimation of formative constructs.
3. *Researcher-determined approach* This category includes arbitrary, literature-driven (theory), or consensus-based weights. The use of these approaches seems to be supported by the opinion of experts, according to whom data analysis is neither needed nor appropriate to decide how to combine indicators in certain models, and the importance of indicators must be defined not by the data but by the objectives of the researchers developing the instrument [12].
  In fact, an approach proposed in the literature to deal with the problem of parameter estimation in SEM is to predetermine the contribution of the indicator to the construct [γ in Eq. (3)] [23]. Experts have suggested that weights could be determined a priori, according to the theoretical contribution of the indicators to the construct [23, 68].
  According to Cadogan et al., if there is no theory suggesting the contrary, formative indicators should have equal weightings [36]. For example, in the earlier version of the Human Development Index, which combined three areas (longevity, educational attainment, and standard of living), researchers intentionally gave equal weights to each one of the aspects [69].
  All these recommendations are in keeping with the underlying theory, as Lee states: “a formative variable is simply a researcher-defined composite of subdimensions, and testing these models is unnecessary” [67].
4. *Mixed approaches*
  - Impact or relevance: Indicators related to symptoms may have particular implications due to individual differences in disease expression and the impact that each symptom can have [38]. Hirsch et al. evaluated the impact or relevance of symptoms using logistic regression analysis [70]. In brief, respondents to a disease screening survey underwent a physical exam and had a battery of disease-related tests. Clinical experts, blinded to the responses to the survey, rated each patient’s probability of having the disease by assessing their test results. The experts’ responses were combined using Bayesian methods. Individuals with 50 % or higher probability of disease were considered disease positive, whereas the remaining patients were considered controls. A logistic regression model was then used to obtain weights that reflected the importance of each question to predict the outcome, allowing calculation of weighted scores [70].
  - Another approach that incorporated the importance of a domain to the measurement of QoL was proposed by Hsieh. Conceptualizing QoL from a formative perspective, he proposed a variation of simple multiplicative weights for patient-reported outcomes that included both importance and satisfaction scores [71]. However, there is evidence that this strategy may not be superior to unweighted schemes [72], in keeping with the idea that the responses of an individual to indicators measuring satisfaction already include an implicit estimation of the importance of the indicator to the subject [73].

## Discussion

More than a century ago, the pioneer work of Charles Spearman on correlation methods in the study of intelligence established the foundations of CTT and factor analysis [5, 74].

Classical test theory, at the heart of traditional psychometrics, is the foundation of reflective measurement models. It focuses on the observed scores, which are considered to reflect true scores plus random error [75]. Item-response theory is a family of contemporary psychometric methods that seek to explain or predict the performance of an item or indicator as a function of an underlying latent variable or construct [76]. Despite the differences between CTT and IRT, they share some principles—the observable measures (i.e., indicators) are a function of an underlying construct, variation in the latter precedes variation in the former [23], and all the measures of an instrument share “one and only one” underlying construct [77]. Homogeneity of indicators is a desired property, and statistical methods are used to evaluate this property [12]. The reflective measurement model is based on these principles [78].

Decades ago, however, researchers in sociology recognized that not all constructs can be measured with positively intercorrelated indicators, thus laying the foundations for formative models. These models were later extrapolated to other social sciences, and the theoretical and empirical aspects of formative and reflective measurement models continued to develop [35].

The evolution of concepts explained above shows that the problems and concerns regarding the adequacy of the traditional measurement approach are common to a number of research fields. However, although the theory underlying formative measurement models has reached clinical research, it is not widely known. Indeed, the formative approach is seldom used in applied medical research despite the fact that many measurements in this field can be conceptualized as composite indexes.

An important task for the clinical researcher developing a measurement instrument pertains to the choice of a measurement model. This choice is dependent on the ontology (this is, the nature of being or existence) of the underlying construct [27].

From an ontological point of view, if the construct is assumed to exist independent of measurement, it corresponds to the school of philosophical realism, which states that reality is independent of our conceptual schemes or perceptions. On the other hand, if the construct is considered a construction of the human mind and does not necessarily exist independent of measurement, it corresponds to philosophical constructivism [18], in which “the truth is what we create to better negotiate the world of our experience” [79]. Whereas in the reflective model, ascribed to realism, a construct determines its indicators, in the formative model, which is closer to constructivism, constructs are understood to be a summary of the indicators [18].

For example, the construct *anxiety* is measured as a real entity using correlated questions in the 10-item Anxiety Symptom Scale (i.e., reflective measurement model), and the construct g*ender inequality* is measured using the Gender Inequality Index, a researcher-created tool composed of heterogeneous indicators such as reproductive health, empowerment, and labor market participation (i.e., formative measurement model). A formative measurement is therefore seen as a theoretical entity that is not real beyond what is defined by the indicators, and that does not exist independent of its measurement [11].

Since the goal of a measurement instrument is to provide a score by combining the values of its indicators, the considerations surrounding the nature of indicators are critical to the result of a measurement tool. In general, it can be said that, whereas reflective measures can be handled by simple summation, formative measures benefit from the use of weighted scores that preserve the contribution of each of the aspects of the construct. We have reviewed different approaches to obtain weights as a means to preserve the relevance of each indicator.

Each of the techniques described here has advantages and disadvantages, and the choice of a weighting method should rest on contextual factors.

There are limitations to our study that must be pointed out. We limited our search to the terms “formative” and “reflective”, since the inclusion of the terms “causal/cause/effect”, which are commonly used the English language, resulted in the retrieval of a great quantity of irrelevant publications. However, the references of the retrieved articles were hand-searched in order to find related and relevant literature.

The present study attempts to disseminate measurement concepts introduced in health research by the work of investigators such as Feinstein, de Vet, Fayers, and Hand [22, 37, 80], while also offering essential concepts in measurement that would allow the healthcare practitioner to better appraise and understand the measurement tools that are used in everyday clinical assessment.

In an era when medicine is centered on the measurement of clinical outcomes [81], with the assessment of patient satisfaction, quality of care, and efficient use of resources providing the evidence that drives modern health care systems [82], the present work was deemed timely and relevant.

## Conclusion

In conclusion, it is important for the clinical researcher to be familiar with the differences between reflective and formative measurement models, including the different approaches to obtaining a summary score. Summary scores are an integral part of the validity of a measurement tool. Whereas simple summation is a theoretically sound scoring method in reflective models, formative models likely benefit from a weighting scheme that preserves the contribution of each aspect of the construct.

## Additional files

10.1186/s13104-015-1561-6 Search strategies.
10.1186/s13104-015-1561-6 Study selection, flow diagram.

## Abbreviations

CTT: classical test theory
SES: socio-economic status
SEM: structural equation modeling
IRT: item-response theory
QoL: quality of life
SEIQoL: schedule for the evaluation of individual quality of life
MIMIC: multiple indicator multiple cause
RS: rating scale
TTO: time trade-off
SG: standard gamble
