# Supplementary material for: A critical review of scoring options for clinical measurement tools
Source: BMC Res Notes. 2015 Oct 28;8:612. doi: 10.1186/s13104-015-1561-6 (PMC4624594; doi:10.1186/s13104-015-1561-6)
Supplement: Supplementary file 1 — 10.1186/s13104-015-1561-6 Search strategies. [file 13104_2015_1561_MOESM1_ESM.docx]

| **Database** | **Search Strategy** |
| --- | --- |
| OvidSP MEDLINE (1946 to February 25, 2013) | ((formative or reflective) adj2 (model* or measure* or indicator*)).ti,ab. |
| OvidSP EMBASE (1947 to 2013 Week 08) | ((formative or reflective) adj2 (model* or measure* or indicator*)).ti,ab. |
| OvidSP PsycINFO < 1967 to February Week 3 2013 | ((formative or reflective) adj2 (model* or measure* or indicator*)).ti,ab. |
| EBSCOHost CINAHL 1982 to February 25, 2013 | (TX (formative OR reflective) N2 (model* or measure* or indicator*)) |
| ProQuest ABI/INFOR 1960 to February 25, 2013 | AB,TI (formative NEAR2 model*) OR AB,TI (formative NEAR2 measure*) OR AB,TI (formative NEAR indicator*) OR AB,TI (reflective NEAR2 model*) OR AB,TI (reflective NEAR2 measure*) OR AB,TI (reflective NEAR2 indicator*) |
