# Supplementary material for: A critical review of scoring options for clinical measurement tools
Source: BMC Res Notes. 2015 Oct 28;8:612. doi: 10.1186/s13104-015-1561-6 (PMC4624594; doi:10.1186/s13104-015-1561-6)
Supplement: Supplementary file 2 [file 13104_2015_1561_MOESM2_ESM.docx]

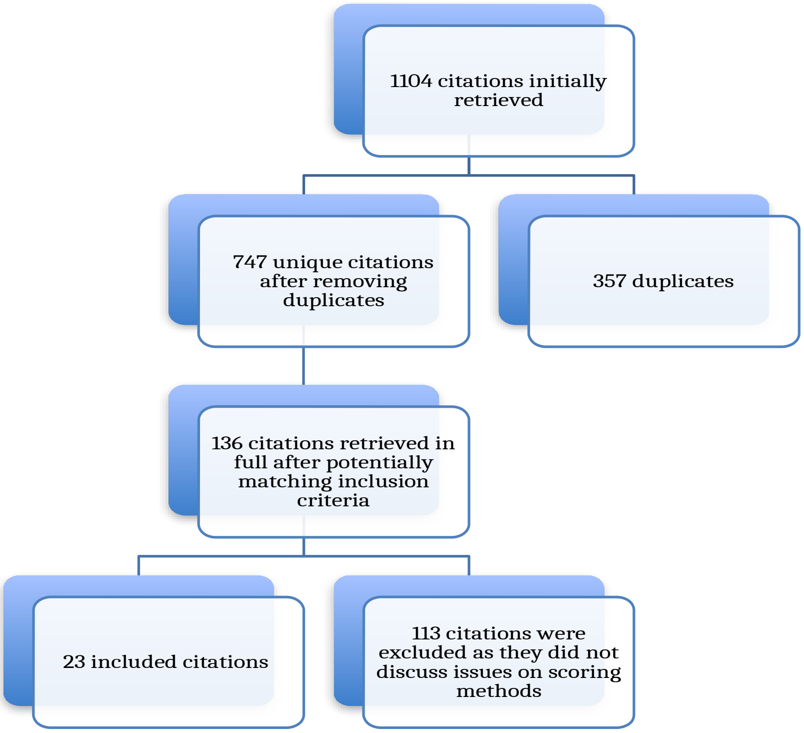
1104 citations initially retrieved
747 unique citations after removing duplicates
357 duplicates
136 citations retrieved in full after potentially matching inclusion criteria
23 included citations
113 citations were excluded as they did not discuss issues on scoring methods
